# Treatment of Xerostomia with Mesenchymal Stem Cells – A Systematic Review and Meta-Analysis of Clinical Trials

**DOI:** 10.1007/s12015-026-11105-9

**Published:** 2026-03-20

**Authors:** Joachim Hansen, Amanda-Louise Fenger Carlander, Kathrine Kronberg Jakobsen, Josephine Skjoldbirk, Christian Grønhøj, Christian von Buchwald

**Affiliations:** Department of Otorhinolaryngology, Head and Neck Surgery and Audiology, Rigshospitalet, University of Copenhagen, Inge Lehmanns Vej 7, section 7046, Copenhagen, 2100 Denmark

**Keywords:** Mesenchymal stem cells, Xerostomia, Sjögren’s Syndrome, Radiation, MSC, Clinical trial

## Abstract

**Background:**

Salivary hypofunction and xerostomia are major complications for overall quality of life. Two of the most frequent causes of xerostomia are radiotherapy of the head and neck and Sjögren’s disease. An increasing number of clinical human studies suggest that mesenchymal stem cell (MSC) therapy can ameliorate symptoms of xerostomia. However, a meta-analysis is yet to summarize the results. The primary outcome of this study was unstimulated salivary flow rate (UWS) after treatment with MSCs.

**Methods:**

The MEDLINE, EMBASE, and Cochrane databases were searched for eligible studies. Eligible studies were: clinical studies including patients with salivary hypofunction due to either radiotherapy or Sjogren’s disease who were subsequently treated with MSCs. A meta-analysis was conducted for the included randomized controlled trials. Secondary outcomes include method of administration, number of MSC used, change in patient reported outcomes, development of drug-specific antibodies, and safety.

**Results:**

Eight studies were included describing 5 clinical trials. 230 participants were treated, hereof 126 received MSC treatment. In the meta-analysis, an increase in UWS of 0.06 mL/min (95%CI: -0.05 to 0.17) were found. In a subgroup analysis of radiation induced xerostomia, a significant increase in UWS of 0.03 mL/min (95%CI: 0.01 – 0.05) were found. All trials reported improvement in patient reported outcomes. Further, no treatment-related serious adverse events were reported, and few, minor, and temporary adverse events was observed.

**Conclusion:**

MSC therapy for xerostomia showed a potential but modest benefit in improving salivary gland function. Further, MSC treatment was found to be safe with minor, temporary adverse events.

**Supplementary Information:**

The online version contains supplementary material available at 10.1007/s12015-026-11105-9.

## Introduction

Xerostomia, defined as the subjective sensation of dry mouth, and salivary hypofunction are associated with substantial morbidity and negatively affect quality of life across social and occupational domains [1, 2]. Common manifestations include impaired oral functions such as speech, mastication, and swallowing as well as increased risk of dental caries, and reduced sleep quality [3, 4].

Radiation therapy targeting the head and neck region and Sjögren disease (SjD) are among the most prevalent etiologies of xerostomia [2]. Salivary glands exhibit high radiosensitivity and are susceptible to radiation-induced injury, which can result in both acute and chronic responses. These include inflammation, interstitial fibrosis, and loss of acinar cells, salivary gland stem cells, and blood vessels, leading to hyposalivation and xerostomia [5, 6]. The prevalence of moderate to severe xerostomia after head and neck radiation has been reported to range from 41 to 85% [7, 8]. The global incidence of head and neck cancer is increasing [9–11], suggesting a growing need for effective treatments. SjD is a chronic autoimmune disease affecting the exocrine glands, particularly the lacrimal and salivary glands, and is characterized by lymphocytic infiltration of the exocrine glands inducing inflammation, interstitial fibrosis, and loss of acinar cells, leading to hyposalivation [12]. Hence, despite two different etiologies to xerostomia, SjD and radiation of the head and neck share many of the pathophysiologic changes leading to xerostomia. SjD affects around 0.05% to 0.5% of the populaion in Dnmark [13, 14], predominantly women (approximately 90%).

Currently, available therapies for xerostomia are limited to symptomatic management and are largely inadequate in effect and duration [15, 16]. This highlights the need for a new treatment strategy. Mesenchymal stem cells (MSCs) have emerged as a promising candidate due to their immunomodulatory, anti-inflammatory, and regenerative properties [17]. Specifically, in a meta-analysis of MSC treatment in the salivary glands for in vivo models, MSC were observed to reduce fibrosis, restore acinar structure, mitigate apoptosis and secrete anti-inflammatory cytokines in the salivary glands [18], linking the effect to the pathophysiologic changes induced by both SjD and radiotherapy of the head and neck. MSCs reside in almost all connective tissues including bone-marrow and adipose tissue [19]. Several preclinical studies have been conducted to investigate the efficacy of MSC therapy for the treatment of xerostomia. A recent meta-analysis of preclinical in vivo models of radiation-induced xerostomia demonstrated a significant improvement in unstimulated salivary flow rate (UWS) following MSC therapy as well as a favorable safety profile [18], supporting continued investigation in clinical trials. An increasing number of clinical studies suggest MSC therapy in humans may enhance salivary flow rate and ameliorate xerostomia-related symptoms [20–24]. However, existing studies have limited sample size, and a meta-analysis of clinical trials has yet to summarize the results. Therefore, in this systematic review and meta-analysis, the effects of MSC treatment for salivary hypofunction due to radiotherapy in the head and neck or SjD will be investigated.

## Objectives

The primary objective was the efficacy of MSC treatment compared to placebo, measured as UWS by sialometry. Further, the secondary objectives were to evaluate differences in stimulated salivary flow rate (SWS), patient reported outcome measures, methods of intervention administration, number of injected MSCs, development of de novo drug-specific antibodies (DSAs), and safety.

## Methods

### Protocol and Registration

This systematic review and meta-analysis have been reported following the PRISMA 2020 statement. The protocol has been published elsewhere [25] and uploaded to PROSPERO prior to the literature search and data analysis. PROSPERO ID: CRD42024527183.

The original protocol investigated the difference in efficacy of adipose-derived MSC (ASC) vs. bone marrow derived MSC (BM-MSC) treatment for hyposalivation and xerostomia. However, after full text screening only one trial, with no control group, investigating BM-MSC treatment were found. Hence, a meta-analysis could not properly investigate the primary objective. Therefore, prior to data extraction, the primary objective was changed to investigate all types of MSC treatment (including both ASC and BM-MSC treatment) for hyposalivation and xerostomia. This modification comes with a risk of the search strategy not fitting the new objectives. However, it was assessed that the search strategy appropriately fitted to the new primary outcome. Further, a network meta-analysis was no longer appropriate. All other items in the protocol were still followed. Prospero was not updated after modification of the primary objective, as protocol alterations was to be disclosed in the final article, as stated in the published protocol. However, this represents a deviation from best systematic review practices.

### Eligibility Criteria

Studies were eligible for inclusion if they described clinical trials including human participants, suffering from xerostomia due to Sjögren’s disease or following radiotherapy for a head and neck cancer. Further, the intervention of interest was MSC treatment, irrespective of the method of administration. Lastly, the studies must have reported the UWS before and after treatment.

### Search Strategy and Information Sources

The following databases were searched for eligible studies: MEDLINE, EMBASE, and the Cochrane CENTRAL database of Controlled Trials. Grey litterateur and clinicaltrials.gov were not included in the search. Studies were included from the inception of the databases.

The search phrases used in this study has been peer reviewed according to the PRESS criteria [26] by an information specialist affiliated with the institution of the corresponding author. The search phrases can be found in the supplemental materials. No limits or restrictions were used in the search.

### Data Management

The online software Covidence © (Covidence systematic review software, Veritas Health Innovation, Melbourne, Australia. Available at www.covidence.org.) was used to import literature searches, remove duplicates, screen the imported articles, and extract data. All statistical analysis were done in the software R-studio© and Cochranes Review Manager.

### Selection and Extraction Process

All identified articles from the search were screened by title and abstract by two authors. JH screened all articles, AMFC screened articles from the initial search, and JS screened articles from the updated search. The subsequent full-text screening was conducted by JH. Any discrepancies from the screening between the reviewers were resolved by consensus.

Data was extracted from each included study after the screening process and put in standardized forms developed a priori. Data was extracted by JH and JS. Any discrepancies were resolved by consensus.

Data from graphs was collected with the software WebPlotdigitizer©.

## Outcome Measures

### Primary outcome

The primary outcome was the UWS rate after intervention.

The follow-up period varies between trials. Therefore, this outcome was a priori divided into two groups:

1. Short-term response up to 6 months after treatment. The time point closest to 4 months will be prioritized.
2. Long-term response from 6 months to 2 years. The latest time point will be prioritized.

The above timepoints were chosen based on studies investigating MSC treatment for xerostomia the authors were aware of before conducted this study [21–23, 27].

### Secondary outcomes

Defined a priori secondary outcomes were: change in stimulated salivary flow rate, change in patient reported outcome measures (PROMs), method of administration, development of drug-specific antibodies, number of injected MSCs, and safety evaluated as adverse events.

### Assessment of Risk of Bias

Risk of bias was assessed with the tool ROBINS-I [28] for the included non-randomized trials, and included randomized controlled trials were assessed with the tool RoB-2 [29], both tools developed by Cochrane. In a post hoc analysis, publication bias was assessed using the ROB-ME tool developed by Cochrane [30].

### Data analysis

A descriptive synthesis was conducted for all included studies. Randomized controlled trials were included in the meta-analysis. The effect estimate for the primary outcome was the mean difference in UWS, calculated either as the postintervention value or as the change from baseline, depending on the data reported. When possible, postintervention values were converted to change scores. When such conversion was not feasible, change scores and postintervention values were pooled according to the guidance provided in the Cochrane Handbook for Systematic Reviews [31]. The *I*^2^ index and Chi^2^ test for heterogeneity was calculated. If there were signs of heterogeneity, a random effects model adjusted to Hedge’s g was applied. If signs of low heterogeneity, a fixed-effect model was applied. For the analysis on safety, treatment related serious adverse events, and adverse events were pooled and reported as an absolute risk, risk difference, and a relative risk ratio.

It was assessed whether the PROMs could be pooled by searching for evidence of correlation between the PROMs. Further, the validity, responsiveness, and reliability were assessed. If no evidence to support the above assessments was found, it was assessed if the underlying concepts of the different PROMs are the same, needed to be divided in groups, or cannot be measured together.

Subgroup analysis was performed for exposure to the salivary glands (radiotherapy or SS). For analysis of subgroups, a fixed effect or random effects model was used according to signs of heterogeneity, as for the main meta-analysis. The credibility was assessed with the ICEMAN tool [32].

## Results

**Fig. 1:**
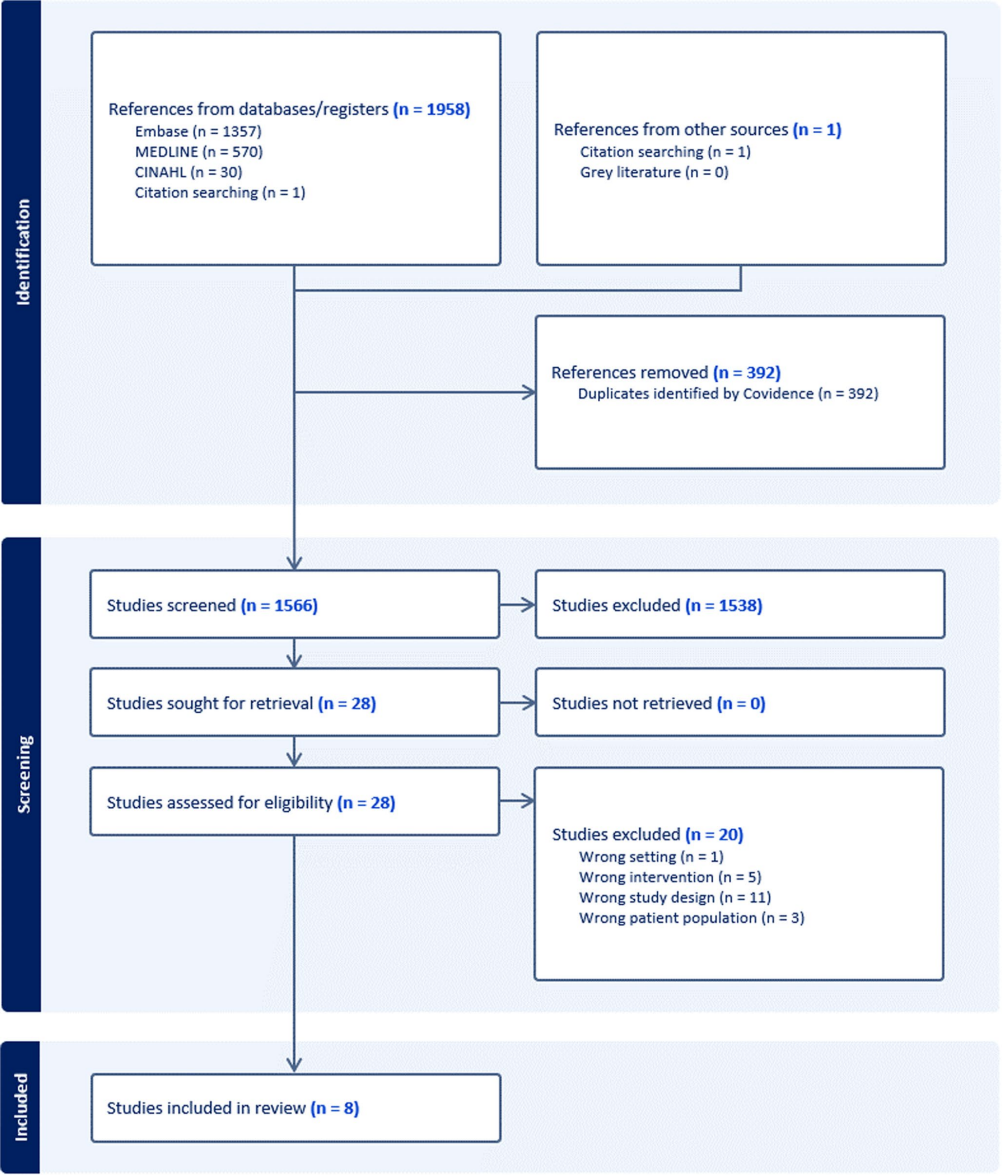
Prisma flowchart

The search was conducted on the 15th of September 2025. A total of 8 studies met the inclusion criteria and were included in this study, as seen in Fig. 1. Of these, 3 studies described long-term follow-up for already included trials. Therefore, 5 different clinical trials were included.

## Description of studies

Three randomized controlled trials and two non-randomized clinical trials without a placebo group were included.

One trial used BM-MSCs while four trials used ASCs. One trial used DMSO as placebo. Two trials used saline water as placebo. All trials used ultrasound-guided injections in the salivary glands as method of intervention. In total 126 participants were treated with MSCs in the included trials. 104 participants were treated with placebo. The follow-up time ranged from 6 to 12 months. The number of MSCs used per treatment differed among the trials. The trial characteristics can be seen in Table 1.

**Table 1:** Characteristics of included studies. RCT: Randomized clinical trial, CT: Clinical trial, SjD: Sjögren’s disease, RIX: Radiation-Induced Xerostomia, ASC: Adipose-derived Mesenchymal Stem cell, BM-MSC: Bone-marrow derived Mesenchymal Stem Cell, SMG: Submandibular gland, PG: Parotid gland

Study ID	Ref	Study design	Number of participants	Type of exposure	Type of MSC	Type of placebo	Follow-up time	Number of MSC	Method of administration
1	Fangfang Li et al. [33]	RCT	64	SjD	Allogeneic ASC	Saline water	1, 3 ,6 months	$5*{10}^{4}$ ASCs per kg of participant in each PG	Ultrasound guided injection
2	G.C. Blitzer et al. [21] **The MARSH trial**	CT	6	RIX	Autologous BM-MSC	No control group	1, 3, 12 months	$10*{10}^{6}$ BM-MSCs in right SMG	Ultrasound guided injection
3	C. Grønhøj et al. [22] **The MESRIX trial**	RCT	30	RIX	Autologous ASCs	Saline water + human albumin	1 and 4 months	$2.8*{10}^{6}$ ASCs * volume of SMG in cm3 in each SMG	Ultrasound guided injection
*4*	C. Lynggaard et al. [34]	12 months follow-up of study ID 3, the MESRIX trial							
5	C. Lynggaard et al. [23] **The MESRIX-II trial**	CT	10	RIX	Allogeneic ASCs	No control group	4 months	$25*{10}^{6}$ ASCs in each SMG. $50*{10}^{6}$ in each PG	Ultrasound guided injection
*6*	K. K. Jakobsen et al. [35]	12 months follow-up of study 5, the MESRIX-II trial							
7	K.K. Jakobsen et al. [27] **The MESRIX-III trial**	RCT	120	RIX	Allogeneic ASCs	DMSO	4 months	$25*{10}^{6}$ ASCs in each SMG	Ultrasound guided injection
*8*	A.L. Carlander et al. [36]	12 months follow-up of study 7, the MESRIX-III trial							

## Efficacy of MSC treatment

Three of the included studies included a placebo group and could therefore be included in the meta-analysis.

**Fig. 2:**
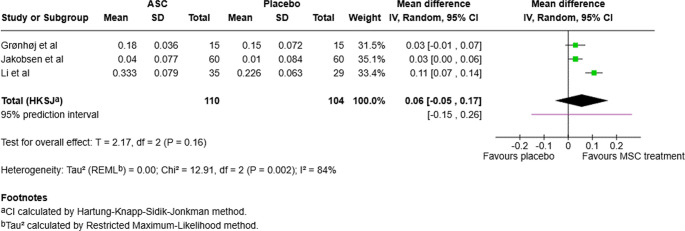
Forest plot for UWS short term effect

For the UWS short-term effect (up to 6 months after treatment), the *I*^*2*^ test for heterogeneity was 84% and p-value for Chi^2^ was 0.002. Therefore, there were signs of high heterogeneity. However, all included studies measured the same outcome using the same method. Hence, a random effects model was used and presented as a mean difference (MD). The MD was 0.06 ml/min (95%CI: -0.05 to 0.17), favoring MSC treatment. The 95% prediction interval ranged from − 0.15 to 0.26. See Fig. 2.

For the UWS long-term effect (6 months to 2 years after treatment), only two studies reported long-term effects. *I*^*2*^ was 44% and the p-value of Chi^2^ was 0.18, indicating low heterogeneity. Therefore, a fixed-effects model was used and presented with a MD. The MD was 0.01 ml/min (95%CI: -0.02 to 0.05), favoring MSC treatment, see Fig. 1 in supplemental materials.

For the SWS short-term effect, *I*^2^ was 88% and the p-value for Chi^2^ was 0.02, indicating high heterogeneity. Therefore, a random-effects model was used and presented as a MD. The MD was 0.05 ml/min (95%CI -0.32 to 0.42), see Fig. 2 in supplemental materials.

For the SWS long term effects, the *I*^*2*^ was 0% and the p-value for Chi^2^ was 0.95 indicating low heterogeneity. Therefore, a fixed-effects model was used and presented with a MD. The MD was − 0.04 mL/min (95% CI: -0.17 to 0.10), favoring placebo, see Fig. 3 in supplemental materials.

**Fig. 3:**
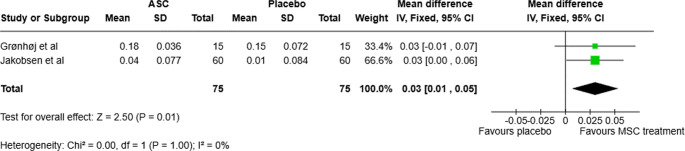
Forest plot for UWS short term effect in patients with radiation-induced xerostomia

A subgroup analysis for type of exposure was made. As there were only one trial which included participants with Sjogren’s disease, only a subgroup analysis for radiation induced xerostomia was made which evaluated change in UWS up to 6 months after treatment. The *I*^2^ was 0% and the p-value for Chi^2^ was 1.0 indicating minimal heterogeneity. Hence, a fixed-effect model was used. The MD was 0.03 (95% CI: 0.01 to 0.05) favoring MSC treatment, See Fig. 3. Further, by using the ICEMAN tool, the subgroup analysis had high credibility. The tool can be seen in the supplemental materials.

### Safety

All 5 included trials reported AEs. In total, 126 patients were treated with MSCs, and 104 patients were treated with placebo. No treatment-related SAEs were observed. 19 patients receiving MSCs and 3 patients receiving placebo were observed to have treatment-related AEs. This corresponds to a risk difference of 12% and a RR of 1.68 (95% CI: 1.4 to 2.1). All AEs were mild, transient, and local. The most common treatment-related AEs were temporary swelling or soreness of the treated glands, hematoma at the injection site, and temporary lymphadenopathy. Nearly all participants had temporary mild to medium pain during treatment. However, this AE is procedure-related. All AEs were minor and temporary. In Table 2, an overview of the AEs in the specific trial can be seen.

**Table 2:** Overview of safety in the included trials

Ref	Treatment related SAEs	Treatment related AEs
Fangfang Li et al. [33]	None of 35 patients	Intervention group: 2/35 patients - 2 patients had temporary itchiness at injection site 2 h after treatment. Control group: 0/29 patients
G.C Blitzer et al. [21]	None of 6 patients	Intervention group: 4/6 patients (66%) - 2 patients had temporary lymphadenopathy - 2 patients had temporary decrease in lymphocytes - 3 patients had temporary grade 1/10 pain after injection. No control group.
C. Grønhøj et al. [22]	None of 15 patients	Intervention group: 0/15 patients Control group: 0/15 patients
C. Lynggaard et al. [34]	None of 10 patients	Intervention group: 1/10 patients - 1 patient had vasovagal syncope during treatment. No control group.
K.K. Jakobsen et al. [35]	None of 60 patients	Intervention group: 12/60 patients - 9 patients had temporary swelling of the submandibular glands - 1 patient had temporary hematoma at the injection site - 2 patients had temporary soreness of the submandibular glands - All patients had temporary pain during injection. Control group: 3/60 patients - 1 patient had temporary hematoma at the injection site - 1 patient had temporary soreness of the submandibular glands - 1 patient had a near-syncope during injection - All patients had temporary pain during injection
In total (excluding pain during injection)	None of 126 patients receiving ASC’s	Receiving ASC: 19/126 (15%) Receiving placebo: 3/104 (3%) Risk difference: 12% RR: 1.68 (95% CI: 1.4 to 2.1)

### Patient Reported Outcomes

In general, the included studies report trends towards improvement of PROMs, but no significant differences were found when comparing intervention and control groups. The exception being the trail by Fangfang li et al., [33] which reported a significant reduction in ESSPRI score 1 months after treatment with MSCs. ESSPRI is a PROM assessing the subjective disease activity of Sjogren’s disease [37]. Thereby, focusing on sicca symptoms as well as fatigue. Three trials reported significant improvements of the included PROMs in the intervention group [22, 23, 27]. Jakobsen et al. further noted that the improvement seen in the EORTC QLQ-H&N35 exceeded changes measured in other studies to be clinically relevant. Grønhøj et al. only found significant improvements in domains regarding thirst. The last trial by Blitzer et al. [21] demonstrated trends toward improvement in the included PROMs, although these did not reach statistical significance.

A few different PROMs were used in the included trials, see Table 3. Unfortunately, no studies have been found supporting the validity, responsiveness, and reliability between the different PROMs. Further, the concepts investigated are too different to be able to pool the diverse PROMs. The Michigan Xerostomia-related Quality of Life (MXeQoL) and the Xerostomia Questionnaire both assess xerostomia and share several overlapping items. However, more than half of the MXeQoL items address the broader impact of xerostomia on quality of life beyond oral and swallowing discomfort. Consequently, pooling these measures is not feasible because they mix different domains. The Xerostomia VAS includes items that overlap with the Xerostomia Questionnaire but uses broader formulations and does not assess sleep, unlike the Xerostomia Questionnaire. Additionally, scoring differs. The Xerostomia VAS rates each item on a continuum from ‘not dry at all’ to ‘very dry,’ whereas the Xerostomia Questionnaire uses a 1–10 scale. Therefore, pooling was not possible. Similarly, the MD Anderson Dysphagia Inventory and the EORTC QLQ-H&N35 share some overlapping items. However, the former focuses exclusively on dysphagia, while the latter also addresses dry mouth and sticky saliva. Thus, these PROMs cannot be pooled. The Xerostomia Questionnaire (XQ) have been used in the RCTs conducted by C. Grønhøj et al. [22] and K. K. Jakobsen et al., [27] but due to differences in the reporting of data, a meta-analysis cannot be performed.

**Table 3:** Patient reported outcomes used

ref	Type pf PROMS
Fangfang Li et al. [33]	ESSPRI
G.C. Blitzer et al. [21]	Michigan Xerostomia related Quality of Life MD Anderson Dysphagia Index
C. Grønhøj et al. [22]	Xerostomia Questionnaire Xerostomia-VAS
C. Lynggaard	EORTC QLQ-H&N35 Xerostomia Questionnaire
K.K. Jakobsen	EORTC QLQ-H&N35 Xerostomia Questionnaire

### Drug-Specific Antibodies

Three of the included trials used allogeneic MSCs. Fangfang Li et al. did not measure the development of DSAs. Lynggaard et al. report that 20% (2/10 patients) of the treated patients developed DSAs after treatment. Jakobsen et al. report that 31.7% (19/60) of the treated patients developed DSAs after treatment. Jakobsen et al. further report that the group who developed DSAs had a mean UWS increase of 15.6% while the group that did not develop DSAs had a mean UWS increase of 50.2%.

### Number of Injected MSCs

The total number of injected MSCs differed among the trials. The trial by K.K. Jakobsen et al. [27] and C. Lynggaard et al. [34] used $25*{10}^{6}$ MSCs per gland. Additionally, C. Lynggaard et al. also treated the parotid gland with $50*{10}^{6}$ MSCs per gland. The trial by G.C. Blitzer et al. [21] used $10*{10}^{6}$ MSCs per. In the trial by Fangfang Li et al. used $\mathrm{50,000}\frac{MSCs}{kg\;of\;the\;patient}$. If assuming a patient weight of 80 Kg, a total number of $4*{10}^{6}$ MSCs per gland was used. In the trial by C. Grønhøj et al., the amount was dependent on the volume of the submandibular gland. The mean volume of the submandibular glands in the trial by C. Grønhøj et al. was 7.8 $m{m}^{3}$, and thereby the mean number of injected MSCs per gland was $21.8*{10}^{6}$.

### Risk of Bias Assessment

**Fig. 4:**
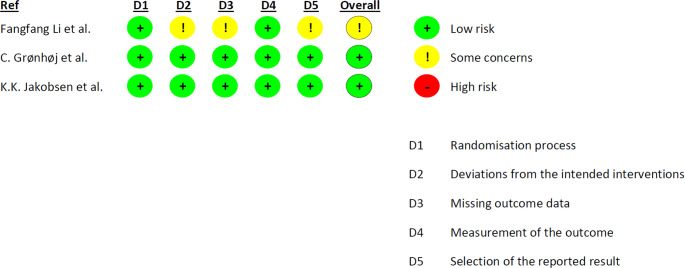
Risk of bias for included RCTs

The tool RoB-2, developed by Cochrane, was used to assess the risk of bias in the included RCTs (Fig. 4). The trials by C. Grønhøj et al. [22] and K.K. Jakobsen et al. [27] was assessed to have low risk of bias in all domains. The trial by Fangfang Li et al. [33] were assessed to have some concerns in risk of bias overall and in the domains of deviations from the intended interventions, missing outcome data, and measurement of the outcome, due to a lack of information in the respective domains.

**Fig. 5:**
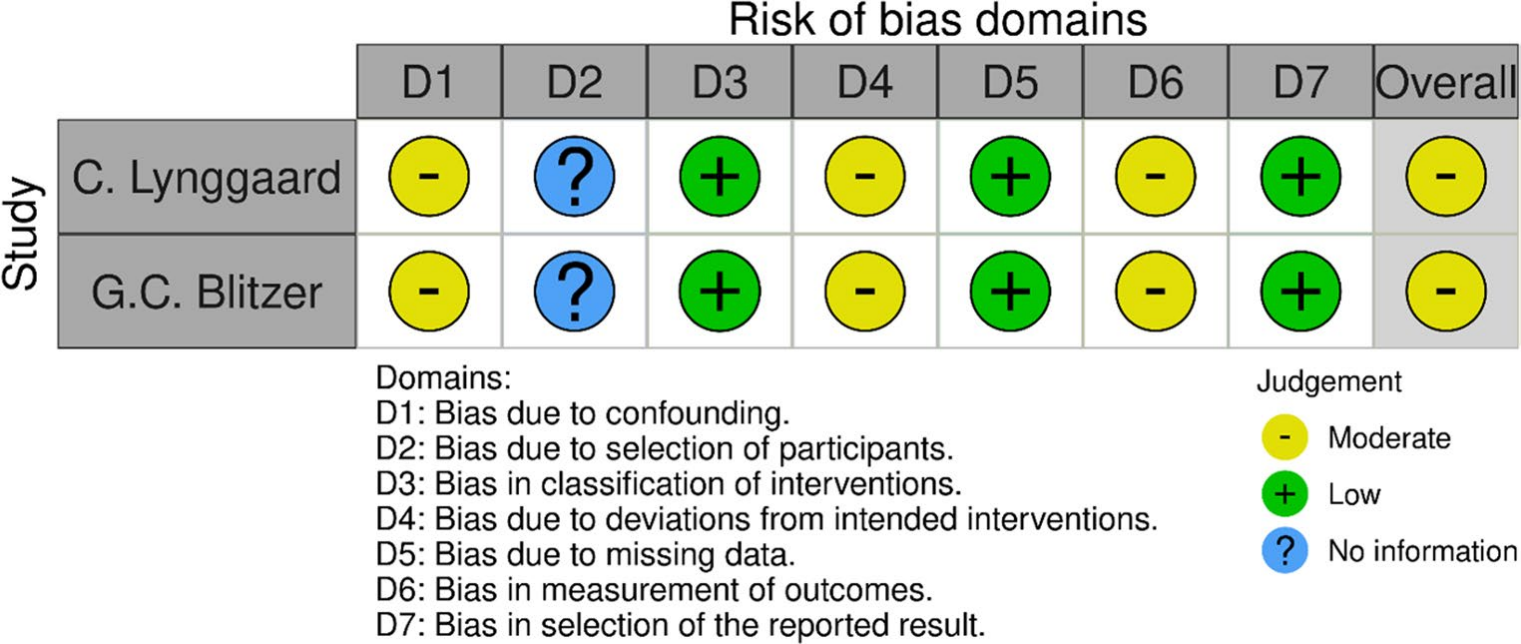
Risk of bias for included non-randomized trials

The tool ROBINS-I was used to assess risk of bias in the included non-randomized trials (Fig. 5). Both trials [21, 23] were assessed to have a moderate risk of bias overall, and in the domains of bias due to confounding, bias due to deviations from intended interventions, and bias due to measurements of outcomes. Both trials did not consider confounders, and measurement of outcomes were reported inconsistent. None of the trials reported information of the selection of trial participants.

Risk of bias due to missing evidence, publication bias, was assessed by the ROB-ME tool. An assessment was done for all five meta-analyses included in this review. All meta-analyses were judged to have low risk of bias. If any reporting bias was present, it is predicted to favor the experimental treatment (MSC treatment). The ROB-ME assessment is included in the supplemental materials.

## Discussion

This is, to the best of our knowledge, the first systematic review and meta-analysis to investigate the effect of MSC therapy on saliva gland function in patients with xerostomia due to Sjögren’s disease and after radiotherapy of a head and neck cancer.

Recent epidemiological data show that late xerostomia and hyposalivation remain common long after radiotherapy [38], emphasizing the limited benefit of current symptomatic treatments. This is also seen in SjD [12]. This persistent burden supports the need for regenerative strategies. MSC therapy may address this gap by targeting underlying glandular injury rather than only alleviating symptoms.

MSC therapy demonstrated a tendency to improve salivary gland function after intraglandular MSC treatment, measured as UWS short-term (up to 4 months) after treatment. However, the results were not significant, and the CI and prediction intervals reveal high uncertainty of the results. Further, the long-term results at 12 months also demonstrated a non-significant increase in UWS after MSC treatment, but the increase in UWS declined from short-term to long-term follow-up, indicating that the effects of MSC treatment is not sustained. Although mesenchymal stem cells (MSCs) are considered immune evasive, they are not immune privileged and therefore have a relatively short lifespan, primarily due to apoptosis induced by immune cells [39]. Hence, repetitive treatments might be needed. These findings align with other trials investigating repeated treatment with MSCs. These trials found that repeated MSC treatments are superior in both objective and subjective measurements [40–50]. However, no trial has yet investigated repeated MSC treatment for xerostomia.

In the subgroup meta-analysis of radiation-induced xerostomia, MSC therapy demonstrated a significant improvement in UWS of 0.03 mL/min (95% CI: 0.01 to 0.05) at four months after treatment. This corresponds to an improvement of 23% in UWS compared to the mean UWS before treatment in the trial by Jakobsen et al. Hence, a relatively small improvement is found. However, the minimal important difference in UWS has yet to be determined. This is currently under investigation in the MESRIX-more trial. Moreover, only two trials are included in the subgroup meta-analysis. Hence, results should be interpreted with caution. Given the relatively modest improvement in outcomes, consideration of spontaneous salivary gland recovery is warranted. Evidence regarding recovery of salivary gland function after head and neck radiotherapy is conflicting [51]. However, reports indicate that a minority of patients experience spontaneous recovery up to 18 months after radiotherapy [51]. The trials included in this analysis enrolled participants with a minimum follow-up of 24 months. Therefore, further spontaneous recovery after intervention is unlikely to have influenced the results. In contrast, patients with SjD are unlikely to experience spontaneous recovery because the condition is progressive [52].

The assessment of PROMs revealed the same tendencies as for the objective outcomes. Hence, tendencies toward fewer symptoms from xerostomia were found. One study found a significant improvement in the ESSPRI-score between the intervention and control groups [33]. However, the ESSPRI-score also includes questions regarding fatigue, a prominent feature of Sjögren’s disease, and does therefore not only include xerostomia-related symptoms. Moreover, three trials found significant improvements in the intervention group [22, 23, 27]. Jakobsen et al. further noted that the improvement seen in the EORTC QLQ-H&N35 exceeded changes measured in other studies to be clinically relevant. Jakobsen et al. used DMSO as placebo, which is also known to have inflammatory effects [53]. This could have influenced the results, as the placebo group also had an improvement in PROMs. The direction of change observed in PROMs was consistent with the direction of change in salivary flow rate. However, because a minimal important difference has not been established for salivary flow rate, it is not possible to determine whether the magnitude of improvement in salivary flow rate corresponds to the magnitude of improvement in the PROMs.

MSC treatment was observed to be safe with no reported treatment-related SAEs. A relative risk of 1.68 was observed in the treatment-groups compared to the placebo groups. However, all reported AEs were mild, localized and temporary (1–2 weeks). The most common procedure-related AE was pain during intraglandular injection of treatment. The most common treatment-related AE was temporary swollen glands. Treatment-related AEs occurred only in the intervention groups, whereas procedure-related AEs occurred in both the intervention and control groups, likely accounting for the difference in AE rates between groups. However, it differed what type of AEs were reported in the trials. This could be due to a different registration of AEs, which results in reporting bias. Nonetheless, these findings align with other thorough meta-analysis on the safety of MSC treatment [54].

SWS was also observed to increase after MSC treatment, but less compared to UWS. This could be due to the glands treated. The parotid glands were treated in one trial [33], the submandibular glands were treated in three trials [21, 22, 27], and one trial treated both the submandibular and parotid glands [23]. The parotid glands are known to produce more stimulated saliva, and the submandibular glands produce more unstimulated saliva [55]. However, the tendencies of the SWS followed those of UWS.

MSCs are known for their lack of MHC class II expression and low levels of MHC class I [56]. These attributes enable allogeneic MSC transplantation. Three of the included trials utilizes allogeneic MSCs and two trials reports observations regarding DSAs. In both trials, a considerable proportion of the treated patients develop DSAs, and Jakobsen et al. further report that UWS increases less in patients who developed DSAs compared to the group who did not develop DSAs after treatment. The reduced efficacy of MSC therapy is reported to result from the shorter lifespan of transplanted MSCs, likely due to a heightened immune response mediated by DSAs [57]. The trials report no AEs caused by the development of DSAs. However, a recent meta-analysis on alloreactive immune response associated with human MSC treatment concludes that the formation of de novo DSAs did not correlate with safety and tolerability, and did not have clinical significance [58]. Further, there is no evidence indicating why some patients develop DSAs. It could be speculated that factors from both the donor of MSC and the patients influence the response. However, this has not yet been investigated. Therefore, there is a need for more thorough investigations of the factors leading to the de novo formation of DSAs and their possible effects. This could be achieved by collecting clinical data on donors of MSC and routinely reporting the results in trails investigating allogeneic MSC treatment. Future trials investigating allogeneic MSC treatment should consider investigating a predictive biomarker for response, as this could be used to choose the patients with most benefit from MSC treatment. Further, stratification for preformed DSAs could be utilized to minimize the effect of DSAs.

The number of injected MSCs per gland differed from $4*{10}^{6}$ to $25*{10}^{6}$ among the included trials. Unfortunately, no pattern is observed in the dose used and the results. Interestingly, the trial using the lowest number of injected MSCs had the largest increase in UWS [33].However, this pattern has not been observed before and therefore needs to be confirmed in other studies. Hence, no consensus can yet be made regarding the optimal number of MSCs used for treatment.

One trial used BM-MSCs [21] for treatment, and four trials used ASCs for treatment [22, 23, 27, 33]. Studies have shown that although ASCs and BM-MSCs share morphology, they behave differently. For example, ASCs have been shown to have greater potential for attenuating fibrosis and have a higher potential for angiogenesis [59, 60]. BM-MSCs are shown to have greater chondrogenic and osteogenic capacities [61]. However, these differences have not yet been tested in a clinical setting, and not enough published trials use BM-MSCs for treatment to conduct a network meta-analysis.

The meta-analysis of the short-term primary outcome demonstrated substantial heterogeneity. In contrast, the subgroup analysis indicated minimal heterogeneity. The trial by Fangfang Li et al. was excluded from the subgroup analysis, suggesting that this study may have been the primary contributor to the heterogeneity observed in the overall analysis. Although the standard deviations reported in Fangfang Li et al.’s trial was comparable to those in other studies, the MD was nearly fourfold higher. The reasons for this discrepancy are unclear. Possible explanations include higher baseline UWS values or the inclusion of participants with SjD, representing a different etiology of xerostomia compared with other trials. Alternatively, unreported factors in the study or its protocol may have contributed. Further, due to the high heterogeneity, results should be interpreted with caution. No formal influence analysis was performed.

The risk of bias assessment for missing evidence revealed low risk of bias for all meta-analyses. However, if any bias were present, it was predicted to favor MSC treatment. The risk of bias assessments for the individual trials revealed some concerns / moderate risk of bias in the majority of trials. The remaining had low risk of bias. Thereby, the results of this meta-analysis could be influenced by the risk of bias. However, the direction of the bias and whether it is systematic is not clear. Hence, the risk of bias introduces uncertainty to the results. The nonrandomized trials were open label, introducing potential bias in outcome reporting, particularly for subjective measures. In such settings, participants may rate their improvement more favorably than they would in blinded trials. In general, the identified biases are likely to overestimate the MSC efficacy.

A sensitivity analysis was not feasible. One option was to exclude open label trials. However, as the open label trials included in this study did not include a control group, they were already excluded from the meta-analyses. Further, the only trial including patients suffering from SjD (Fangfang Li et al. [33]) could be excluded, but this was already done in the subgroup analysis for radiation induced xerostomia. If the subgroup analysis is treated as a sensitivity analysis, the results showed the same tendency as for the main analysis, but the results were significant, adding robustness to the main analysis.

This study has several limitations and strengths. In the meta-analyses for the long-term outcomes, only two trials were included introducing more uncertainty of the results. Moreover, the included non-randomized trials did not describe possible confounders. Confounders include time since radiotherapy, dose of radiotherapy, baseline UWS, and severity of SjD. Both trials were phase 1 safety trials. Therefore, the results must be interpreted with caution. This study is further limited by the low number of trials included, which results in wide confidence intervals in the meta-analysis. Further, the primary outcome was changed after publication of the protocol. However, the change was issued to better suite the included studies and was done prior to data extraction. It was further assessed that the search strategy properly fitted the new objective. This study did not include grey litterateur or clinicaltrials.gov registrations. Hence, any upcoming trials investigating MSC therapy for xerostomia, were not included. However, due to analyzing the results of the included trials, any grey litterateur or clinicaltrials.gov registration would solely be used in a descriptive timeline of future trials. Moreover, both after intervention and changes scores were pooled in the meta-analyses due to inability of converting all to the same score. However, Cochrane state, that there is no statistical reason for not doing so [31] Lastly, title and abstract screening were performed by two authors, but full text screening were performed by one reviewer, introducing potential selection bias. This study also has multiple strengths. The meta-analysis is conducted on the basis of a published comprehensive protocol, the search strategy is peer reviewed, and a ICEMAN analysis for credibility of the subgroup found high credibility. Moreover, all trials included in the meta-analysis were conducted based on published protocols. Further, all studies use the same administration route, which enhances generalizability.

In conclusion, MSC therapy shows signals of modest benefit in improving salivary gland function, with significant effects observed only in patients with radiation-induced xerostomia. The absolute changes were small, and their clinical importance remains uncertain. Further, moderate risk of bias in some studies, high heterogeneity, and low sample size introduces uncertainty of the results. MSC treatment appears safe with only mild and transient adverse events. Larger, well-designed trials with longer follow-up are needed to confirm efficacy and determine optimal treatment parameters.

## Supplementary Information

Below is the link to the electronic supplementary material.

Supplementary Material 1 (DOCX 35.7 KB)

Supplementary Material 2 (DOCX 270 KB)

Supplementary Material 3 (DOCX 112 KB)

## Data Availability

All data, including tables, can be acquired by mail from the corresponding author.
