# Supplementary material for: Treatment of Xerostomia with Mesenchymal Stem Cells – A Systematic Review and Meta-Analysis of Clinical Trials
Source: Stem Cell Rev Rep. 2026 Mar 20;22(4):1933–45. doi: 10.1007/s12015-026-11105-9 (PMC13099713; doi:10.1007/s12015-026-11105-9)
Supplement: Supplementary file 1 — Supplementary Material 1 (DOCX 35.7 KB) [file 12015_2026_11105_MOESM1_ESM.docx]

**Instrument to assess the Credibility of Effect Modification Analyses (ICEMAN)**

**in a meta-analysis of randomized controlled trials**

*Version 1.1*

**Title:** **Treatment of xerostomia with mesenchymal stem cells – a systematic review and meta-analysis of clinical trials**

Authors:

^1^Joachim Hansen, M.D., ^1^Amanda-Louise Fenger Carlander M.D., PhD., ^1^Kathrine Kronberg Jakobsen M.D., PhD,^1^Josephine Skjoldbirk Andersen MSc, ^1^Christian Grønhøj M.D., PhD., DMSc., ^1^Christian von Buchwald M.D., DMSc., professor

1: Department of Otorhinolaryngology, Head and Neck Surgery and Audiology, Rigshospitalet, University of Copenhagen, Denmark

Corresponding author:
Joachim Hansen M.D., Email: [Joachim.hansen.01@regionh.dk](mailto:Joachim.hansen.01@regionh.dk); ORCID: 0000-0001-8395-5037
Department of Otorhinolaryngology, Head and Neck Surgery and Audiology, Rigshospitalet, Inge Lehmanns Vej 7, section 7046, zipcode: 2100, City: Copenhagen Ø, Denmark

**Consider the following important instructions informed by common misapplications of ICEMAN in studies using the instrument**

- Complete a separate credibility assessment per each effect modifier (e.g., age, comorbidity, drug dose, etc.), outcome (e.g., mortality, stroke, duration of hospital stay), time-point (e.g., 3 months, 6 months), and effect measure (e.g. relative risk, risk difference).
- Do not apply ICEMAN if the interaction p-value is 0.1 or larger, i.e., provides very little statistical support for the existence of an effect modification (ICEMAN is designed to address the possible claim of an effect modification rather than the claim of no effect modification).
- Response options on the left indicate definitely or probably reduced credibility, response options on the right probably or definitely increased credibility
- Completely unclear should be interpreted as probably reduced credibility.
- To ensure transparency, provide a supporting comment under each question that provides a rationale for the rating.
- To ensure transparency, provide a copy of the completed ICEMAN instrument in the supplement of your article.

| **CREDIBILITY ASSESSMENT** | | | | |
| --- | --- | --- | --- | --- |
| **Essential preliminary considerations to define the possible effect modification of interest** | | | |  |
| State a single candidate effect modifier (e.g., age or comorbidity): Unstimulated Salivary Flow Rate | | | |  |
| Was the effect modifier measured before or at randomization? [ **x**] yes, continue [ ] no, stop here and refer to manual for further instructions | | | |  |
| State a single outcome and time-point (e.g., mortality at 1 year follow-up): Unstimulated Salivary Flow Rate at 4 months follow-up | | | |  |
| State a single effect measure (e.g., relative risk or risk difference): mL/min | | | |  |
| **1: Is the analysis of effect modification based on comparison within rather than between trials?** | | | | |
| [ **x** ] Completely between | [ ] Mostly between or unclear | [ ] Mostly within | [ ] Completely within | |
| *Subgroup analysis or meta-regression comparing overall effects of each individual trial. This is typical for aggregate data meta-analysis.* | *Subgroup analysis or meta-regression with most information coming from overall effects, but some trials providing within-trial subgroup information* | *Most trials providing within-trial subgroup information; or individual participant data analysis that combines within and between trial information* | *All trials providing within-trial subgroup information or individual participant data; and the analysis separates within from between trial information, e.g., meta-analysis of interactions* | |
| Comment: Subgroup analysis were including only one disease entity. Therefore, the overall effects were used in these trials. Hence, there was no need for individual patient data. | | | | |
| **2: For within-trial comparisons, is the effect modification similar from trial to trial?** [ **x** ] Not applicable: no or one within-RCT comparison | | | | |
| [ ] Definitely not similar | [ ] Probably not similar or unclear | [ ] Mostly similar | [ ] Definitely similar | |
| *Effect modification reported for two or more trials and clearly different directions* | *Effect modification not reported for individual trials or too imprecise to tell* | *Effect modification reported for two or more trials, mostly similar in direction, but considerable differences in magnitude* | *Effect modification reported for two or more trials, similar in direction, only some differences in magnitude* | |
| Comment: | | | | |
| **3: For between-trial comparisons, is the number of trials large?** [ ] Not applicable: no between RCT comparison | | | | |
| [ ] Very small | [ ] Rather small or unclear | [ ] Rather large | [ **x** ] Large | |
| *1 or 2 or in smallest subgroup; 5 or less in continuous meta-regression* | *3-4 in smallest subgroup; 6-10 in continuous meta-regression* | *5-9 in smallest subgroup; 11 to 15 in continuous meta-regression* | *10 or more in smallest subgroup; more than 15 in continuous meta-regression* | |
| Comment: | | | | |
| **4: Was the direction of effect modification correctly hypothesized a priori?** | | | | |
| [ ] Definitely no | [ ] Probably no or unclear | [ ] Probably yes | [ **x** ] Definitely yes | |
| *Clearly post-hoc or results inconsistent with hypothesized direction or biologically very implausible* | *Vague hypothesis or hypothesized direction unclear* | *No prior protocol available but unequivocal statement of a priori hypothesis with correct direction of effect modification* | *Prior protocol available and includes correct specification of direction of effect modification, e.g., based on a biologic rationale* | |
| Comment: Both trials have described the direction of the effect modification | | | | |
| **5: Does a test for interaction suggest that chance is an unlikely explanation of the apparent effect modification?** (consider irrespective of number of effect modifiers) | | | | |
| [ ] Chance a very likely explanation | [ ] Chance a likely explanation or unclear | [ **x** ] Chance may not explain | [ ] Chance an unlikely explanation | |
| *Interaction or meta-regression p-value >0.05* | *Interaction or meta-regression p-value ≤0.05 and >0.01, or no test of interaction reported and not computable* | *Interaction or meta-regression p-value ≤0.01 and >0.005* | *Interaction or meta-regression p-value ≤0.005* | |
| Comment: Data available in the meta-analysis | | | | |
| **6: Did the authors test only a small number of effect modifiers or consider the number in their statistical analysis?** | | | | |
| [ ] Definitely no | [ ] Probably no or unclear | [ ] Probably yes | [ **x** ] Definitely yes | |
| *Explicitly exploratory analysis or large number of effect modifiers tested (e.g., greater than 10) and multiplicity not considered in analysis* | *No mention of number or 4-10 effect modifiers tested and number not considered in analysis* | *No protocol available but unequivocal statement of 3 or fewer effect modifiers tested* | *Protocol available and 3 or fewer effect modifiers tested or number considered in analysis* | |
| Comment: Number considered in the protocols | | | | |
| **7: Did the authors use a random effects model?** | | | | |
| [ **x** ] Definitely no | [ ] Probably no or unclear | [ ] Probably yes | [ ] Definitely yes | |
| *Fixed (or common) effect or fixed effects model explicitly stated* | *Probably fixed effect(s) model* | *Probably random (or mixed) effects* | *Random (or mixed) effects explicitly stated* | |
| Comment: Due to low value of I^2^ a fixed effect analysis was used | | | | |
| **8: If the effect modifier is a continuous variable, were arbitrary cut points avoided?** [ ] not applicable: not continuous | | | | |
| [ ] Definitely no | [ ] Probably no or unclear | [ ] Probably yes | [ **x** ] Definitely yes | |
| *Analysis based on exploratory cut point(s), e.g., picking cut point associated with highest interaction p-value* | *Analysis based on cut point(s) of unclear origin* | *Analysis based on pre-specified cut point(s), e.g., suggested by prior RCT* | *Analysis based on the full continuum, e.g., assuming a linear or logarithmic relationship* | |
| Comment: The minimal important difference is yet to be determined | | | | |
| **9 Optional: Are there any additional considerations that may increase or decrease credibility?** (manual section 3.9) [ **x** ] not applicable | | | | |
|  | [ ] Yes, probably decrease | [ ] Yes, probably increase | | |
| Comment:   \| **10: How would you rate the overall credibility of the proposed effect modification?**  The overall rating should be driven by the items that decrease credibility. The following provides a sensible strategy:   - All responses definitely or probably decrease credibility or unclear 🡪 very low - Two or more responses definitely decrease credibility 🡪 maximum usually low even if all other responses satisfy credibility criteria - One response definitely decreases credibility 🡪 maximum usually moderate even if all other responses satisfy credibility criteria - Two responses probably decrease credibility 🡪 maximum usually moderate even if all other responses satisfy credibility criteria - No response options definitely or probably decrease credibility 🡪 high very likely   Place a mark on the continuous line (or type “x” in editable version) \| \| \| \| \|  \| \| --- \| --- \| --- \| --- \| --- \| --- \| \|  \|  \| \| \| \|  \| \|  \| **x** \| \| \| \|  \| \|  \|  \| \|  \|  \| \| \| \|  \| \|  \|  \| \| \| \|  \| \|  \| **Very low credibility** \| **Low credibility** \| **Moderate credibility** \| **High credibility** \|  \| \|  \|  \|  \|  \|  \|  \| \|  \| Minimal to no support for effect modification;  Use overall effect for each subgroup \| Some but insufficient support for effect modification;  Use overall effect for each subgroup but note remaining uncertainty \| Likely effect modification;  Use separate effects for each subgroup but note remaining uncertainty \| Very likely effect modification;  Use separate effects for each subgroup \|  \| \| Comment: \| \| \| \| \| \| | | | | |
