# Supplementary material for: Treatment of Xerostomia with Mesenchymal Stem Cells – A Systematic Review and Meta-Analysis of Clinical Trials
Source: Stem Cell Rev Rep. 2026 Mar 20;22(4):1933–45. doi: 10.1007/s12015-026-11105-9 (PMC13099713; doi:10.1007/s12015-026-11105-9)
Supplement: Supplementary file 3 — Supplementary Material 3 (DOCX 112 KB) [file 12015_2026_11105_MOESM3_ESM.docx]

# Supplemental materials

Title: Treatment of xerostomia with mesenchymal stem cells – a systematic review and meta-analysis of clinical trials

Authors:

^1^Joachim Hansen, M.D., ^1^Amanda-Louise Fenger Carlander M.D., PhD., ^1^Kathrine Kronberg Jakobsen M.D., PhD,^1^Josephine Skjoldbirk Andersen MSc, ^1^Christian Grønhøj M.D., PhD., DMSc., ^1^Christian von Buchwald M.D., DMSc., professor

1: Department of Otorhinolaryngology, Head and Neck Surgery and Audiology, Rigshospitalet, University of Copenhagen, Denmark

Corresponding author:
Joachim Hansen M.D., Email: [Joachim.hansen.01@regionh.dk](mailto:Joachim.hansen.01@regionh.dk); ORCID: 0000-0001-8395-5037
Department of Otorhinolaryngology, Head and Neck Surgery and Audiology, Rigshospitalet, Inge Lehmanns Vej 7, section 7046, zipcode: 2100, City: Copenhagen Ø, Denmark

## Search phrases

#### Cochrane

ID Search

#1 MeSH descriptor: [Stem Cells] explode all trees

#2 MeSH descriptor: [Stromal Cells] explode all trees

#3 MeSH descriptor: [Stem Cell Transplantation] explode all trees

#4 MeSH descriptor: [Cell- and Tissue-Based Therapy] explode all trees

#5 MeSH descriptor: [Secretome] explode all trees

#6 MeSH descriptor: [Exosomes] explode all trees

#7 MeSH descriptor: [Bone Marrow Transplantation] explode all trees

#8 (adipose tissue-derived mesenchymal stem cell*):ti,ab,kw

#9 (adipose derived mesenchymal stem cell*):ti,ab,kw

#10 (allogeneic mesenchymal stem cell*):ti,ab,kw

#11 (ASC):ti,ab,kw

#12 (ADSC):ti,ab,kw

#13 (BMSC):ti,ab,kw

#14 (MSC):ti,ab,kw

#15 ((bone or adipose) NEAR/5 (stem or stromal or cell*)):ti,ab,kw

#16 (bone-marrow stem cell*):ti,ab,kw

#17 (preadipocyte*):ti,ab,kw

#18 (Processed lipoaspirate cell*):ti,ab,kw

#19 (stromal vascular fraction*):ti,ab,kw

#20 #1 OR #2 or #3 or #4 or #5 or #6 or #7 or #8 or #9 or #10 or #11 or #12 or #13 or #14 or #15 or #16 or #17 or #18 or #19

#21 MeSH descriptor: [Saliva] explode all trees

#22 MeSH descriptor: [Salivation] explode all trees

#23 MeSH descriptor: [Salivary Glands] explode all trees

#24 MeSH descriptor: [Xerostomia] explode all trees

#25 MeSH descriptor: [Salivary Gland Diseases] explode all trees

#26 (saliva*):ti,ab,kw

#27 (saliva* NEAR/5 hypofunction*):ti,ab,kw

#28 ((oral or mouth) NEAR/3 dry*):ti,ab,kw

#29 (hyposalivation*):ti,ab,kw

#30 #21 or #22 or #23 or #24 or #25 or #26 or #27 or #28 or #29

#31 MeSH descriptor: [Radiotherapy] explode all trees

#32 MeSH descriptor: [Radiation] explode all trees

#33 MeSH descriptor: [Radiation Injuries] explode all trees

#34 MeSH descriptor: [Chemoradiotherapy] explode all trees

#35 (postradiation*):ti,ab,kw

#36 (radio-induced):ti,ab,kw

#37 (irradiation*):ti,ab,kw

#38 (radiotherap*):ti,ab,kw

#39 (radiation*):ti,ab,kw

#40 #31 or #32 or #33 or #34 or #35 or #36 or #37 or #38 or #39

#41 MeSH descriptor: [Sjogren's Syndrome] explode all trees

#42 (sjogren*):ti,ab,kw

#43 (sicca*):ti,ab,kw

#44 #41 or #42 or #43

#45 #30 and #40

#46 #30 and #44

#47 #45 or #46

#48 #47 and #20

#### EMBASE

1 exp saliva/

2 exp salivation/

3 exp salivary gland/

4 exp xerostomia/

5 exp salivary gland disease/

6 "saliva*".kf,tw.

7 (saliva* adj5 hypofunction*).kf,tw.

8 ((Oral or mouth) adj5 dry*).kf,tw.

9 hyposalivation*.kf,tw.

10 "Oral dry*".kf,tw.

11 "Xerostom*".kf,tw.

12 1 or 2 or 3 or 4 or 5 or 6 or 7 or 8 or 9 or 10 or 11

13 exp radiotherapy/

14 exp radiation/

15 exp radiation injury/

16 exp chemoradiotherapy/

17 postradiation.kf,tw.

18 radio-induced.kf,tw.

19 "irradiation*".kf,tw.

20 "radiation inju*".kf,tw.

21 "radiotherap*".kf,tw.

22 "radiation*".kf,tw.

23 13 or 14 or 15 or 16 or 17 or 18 or 19 or 20 or 21 or 22

24 exp Sjoegren syndrome/

25 morbus sjogren.kf,tw.

26 "sjogren*".kf,tw.

27 "sicca*".kf,tw.

28 24 or 25 or 26 or 27

29 12 and 28

30 12 and 23

31 29 or 30

32 exp stem cell/

33 exp stroma cell/

34 exp bone marrow transplantation/ or exp stem cell transplantation/

35 exp cell therapy/

36 secretome/

37 exosome/

38 "adipose tissue-derived mesenchymal stem cell*".kf,tw.

39 "adipose derived mesenchymal stem cell*".kf,tw.

40 "allogeneic mesenchymal stem cell*".kf,tw.

41 ASC.kf,tw.

42 ADSC.kf,tw.

43 MSC.kf,tw.

44 BMSC.kf,tw.

45 ((bone or adipose) adj5 (stem or stromal or cell*)).kf,tw.

46 "bone-marrow stem cell*".kf,tw.

47 "preadipocyte*".kf,tw.

48 "Processed lipoaspirate cell*".kf,tw.

49 "stromal vascular fraction*".kf,tw.

50 32 or 33 or 34 or 35 or 36 or 37 or 38 or 39 or 40 or 41 or 42 or 43 or 44 or 45 or 46 or 47 or 48 or 49

51 31 and 50

#### MEDLINE

1 exp Stem Cells/

2 exp Stromal Cells/

3 exp Stem Cell Transplantation/

4 exp "Cell- and Tissue-Based Therapy"/

5 Secretome/

6 Exosomes/

7 exp Bone Marrow Transplantation/

8 "adipose tissue-derived mesenchymal stem cell*".kf,tw.

9 "adipose derived mesenchymal stem cell*".kf,tw.

10 "allogeneic mesenchymal stem cell*".kf,tw.

11 ASC.kf,tw.

12 ADSC.kf,tw.

13 BMSC.kf,tw.

14 MSC.kf,tw.

15 ((bone or adipose) adj5 (stem or stromal or cell*)).kf,tw.

16 "bone-marrow stem cell*".kf,tw.

17 "preadipocyte*".kf,tw.

18 "Processed lipoaspirate cell*".kf,tw.

19 "stromal vascular fraction*".kf,tw.

20 1 or 2 or 3 or 4 or 5 or 6 or 7 or 8 or 9 or 10 or 11 or 12 or 13 or 14 or 15 or 16 or 17 or 18 or

21 Saliva/

22 Salivation/

23 exp Salivary Glands/

24 exp Xerostomia/

25 exp Salivary Gland Diseases/

26 "saliva*".kf,tw.

27 (saliva* adj5 hypofunction*).kf,tw.

28 ((oral or mouth) adj5 dry*).kf,tw.

29 "oral dry*".kf,tw.

30 hyposalivation.kf,tw.

31 21 or 22 or 23 or 24 or 25 or 26 or 27 or 28 or 30

32 exp Radiotherapy/

33 exp Radiation/

34 exp Radiation Injuries/

35 exp Chemoradiotherapy/

36 postradiat*.kf,tw.

37 radio-induced.kf,tw.

38 "irradiat*".kf,tw.

39 "radiation inju*".kf,tw.

40 "radiotherap*".kf,tw.

41 "radiat*".kf,tw.

42 32 or 33 or 34 or 35 or 36 or 37 or 38 or 39 or 40 or 41

43 exp Sjogren's Syndrome/

44 morbus sjogren.kf,tw.

45 "sjogren*".kf,tw.

46 "sicca*".kf,tw. 4191

47 43 or 44 or 45 or 46

48 31 and 42

49 31 and 47

50 48 or 49

51 20 and 50

# Figures


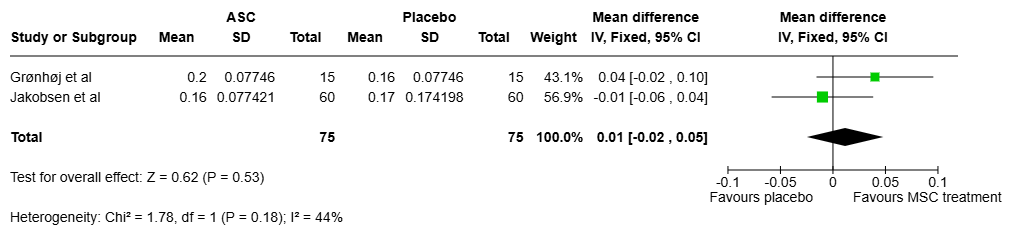


Figure 1 Forest plot for UWS long term effect


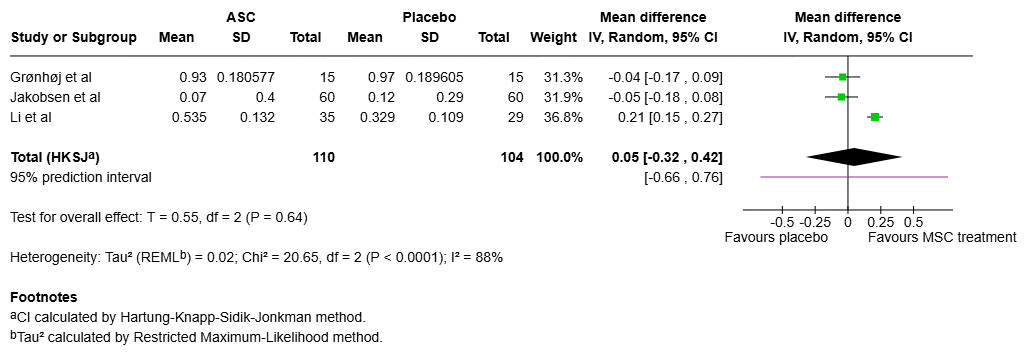


Figure 2 Forest plot for SWS short term effect


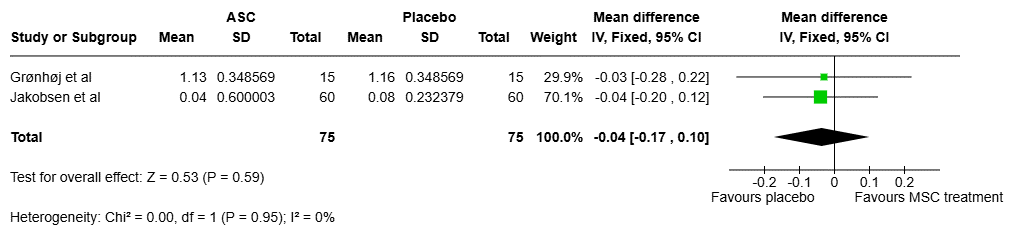


Figure 3 Forest plot for SWS long term effect

**Tool for assessing Risk Of Bias due to Missing Evidence in a meta-analysis (ROB-ME)**

**TEMPLATE FOR COMPLETION**

**Version 1 October 2023**

ROB-ME Development Group:

Matthew J Page, Jonathan AC Sterne, Isabelle Boutron, Asbjørn Hróbjartsson, Jamie J Kirkham, Tianjing Li, Andreas Lundh, Evan Mayo-Wilson, Joanne E McKenzie, Lesley A Stewart, Alex J Sutton, Lisa Bero, Adam G Dunn, Kerry Dwan, Roy G Elbers, Raju Kanukula, Joerg J Meerpohl, Erick H Turner, Julian PT Higgins

**Correspondence to:** Dr. Matthew Page, Methods in Evidence Synthesis Unit, School of Public Health and Preventive Medicine, Monash University, 553 St Kilda Road, Melbourne, Victoria, 3004, Australia. Telephone: +61 9903 0248. Email address: [matthew.page@monash.edu](about:blank)


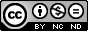


This work is licensed under a [Creative Commons Attribution-NonCommercial-NoDerivatives 4.0 International License](about:blank).

| **Step 1. Select and define meta-analyses that will be assessed for risk of bias due to missing evidence** | | |
| --- | --- | --- |
| **Meta-analysis ID** | **Specify the PICO for all meta-analyses that will be assessed for risk of bias. For example:**  ***Participants:*** *People with shoulder pain*  ***Intervention:*** *Ibuprofen*  ***Comparator:*** *Placebo*  ***Outcome:*** *Pain intensity at short-term (0-12 weeks)*  **Add/delete rows where necessary** | **For each meta-analysis, specify which study designs and results were eligible for inclusion, indicating whether the meta-analysis was restricted to particular:**   - **study designs, and;** - **outcome definitions (e.g. measures, metrics, time points), and;** - **methods of analysis (e.g. analysis populations, crude or adjusted estimates).**   **If such information is reported elsewhere in the systematic review, either indicate the relevant section of the review or copy the information here.**  **For example:**  ***Eligible study designs:*** *Randomized trials*  ***Eligible outcome definitions:*** *Pain scores measured using any scale; up to 12 weeks post-randomization*  ***Eligible methods of analysis:*** *Analyses of change from baseline values; intention-to-treat analysis sample; analyses adjusted for covariates* |
| 1 | **Participants:** Participants suffering from xerostomia due to Sjögrens disease or following radiotherapy for a head and neck cancer  **Intervention:** Mesenchymal Stem Cells  **Comparator:** Placebo  **Outcome:** Unstimulated whole salivary flow rate at short-term (0-6 months) | **Eligible study designs:** Randomized trials  **Eligible outcome definitions:** Unstimulated whole salivary flow rate measured with sialometry up to 6 months after intervention, prioritizing the time-point closest to 4 months  **Eligible methods of analysis:** Change scores and post intervention scores. |
| 2 | **Participants:** Participants suffering from xerostomia due to Sjögrens disease or following radiotherapy for a head and neck cancer  **Intervention:** Mesenchymal Stem Cells  **Comparator:** Placebo  **Outcome:** Unstimulated whole salivary flow rate at long-term (6-24 months) | **Eligible study designs:** Randomized trials  **Eligible outcome definitions:** Unstimulated whole salivary flow rate measured with sialometry 6 -24 months after intervention, prioritizing the latest time-point available.  **Eligible methods of analysis:** Change scores and post intervention scores. |
| 3 | **Participants:** Participants suffering from xerostomia due to Sjögrens disease or following radiotherapy for a head and neck cancer  **Intervention:** Mesenchymal Stem Cells  **Comparator:** Placebo  **Outcome:** Stimulated whole salivary flow rate at short-term (0-6 months) | **Eligible study designs:** Randomized trials  **Eligible outcome definitions:** stimulated whole salivary flow rate measured with sialometry up to 6 months after intervention, prioritizing the time-point closest to 4 months  **Eligible methods of analysis:** Change scores and post intervention scores. |
| 4 | **Participants:** Participants suffering from xerostomia due to Sjögrens disease or following radiotherapy for a head and neck cancer  **Intervention:** Mesenchymal Stem Cells  **Comparator:** Placebo  **Outcome:** Stimulated whole salivary flow rate at long-term (6-24 months) | **Eligible study designs:** Randomized trials  **Eligible outcome definitions:** Stimulated whole salivary flow rate measured with sialometry 6 -24 months after intervention, prioritizing the latest time-point available.  **Eligible methods of analysis:** Change scores and post intervention scores. |
| 5 | **Participants:** Participants suffering from xerostomia following radiotherapy for a head and neck cancer  **Intervention:** Mesenchymal Stem Cells  **Comparator:** Placebo  **Outcome:** Unstimulated whole salivary flow rate at short-term (0-6 months) | **Eligible study designs:** Randomized trials  **Eligible outcome definitions:** Unstimulated whole salivary flow rate measured with sialometry up to 6 months after intervention, prioritizing the time-point closest to 4 months  **Eligible methods of analysis:** Change scores and post intervention scores. |

| **Step 2. Determine which studies meeting the inclusion criteria for the meta-analyses have missing results** |
| --- |
| **For each study meeting the inclusion criteria for one or more of the meta-analyses, assemble available sources of information about the study. This might include the trials register entry (e.g. at ClinicalTrials.gov), study protocol, statistical analysis plan, reports of results of the study (e.g. journal article, clinical study report), or information obtained directly from the study authors or sponsor (e.g. data files supplied).**  **Then compare results available with all available information about what outcomes were measured. If study plans are available (e.g. trials register entry, protocol), compare results available with details of the pre-specified outcomes, to identify any outcomes with no results reported.** **It might be helpful to construct a matrix for each eligible study that lists all outcomes described in the study plans and records whether results were available for each. If no study plans are available, cross-check the methods and results sections against one another to identify any outcomes with no results reported, or results reported incompletely.**  **Then complete the Results Matrix below to indicate (using the symbols in the Key below) whether study results are available for inclusion in each meta-analysis to be assessed for risk of bias.**  **Also specify the total number of participants analysed for an indication of the likely weight of each study in the meta-analysis.**  **Key for Results Matrix**   \| ✓ \| A study result is available for inclusion in the meta-analysis. \| \| --- \| --- \| \| ~ \| No study result is available for inclusion in the meta-analysis, for a reason unrelated to the P value, magnitude or direction of the result. \| \| ? \| Unclear whether an eligible study result was generated. \| \| X \| No study result is available for inclusion in the meta-analysis, likely because of the P value, magnitude or direction of the result generated.  *Optional: Record any information known about the results (if available), such as the direction of effect (e.g. Favours intervention / Favours control), the statistical significance of the result (e.g. P > 0.05), or narrative descriptions (e.g. “No difference”).* \|   **Results Matrix (add/delete rows and columns where necessary)**   \| **Study ID*** \| **Source(s) used** \| **Number of participants analysed**** \| **Result available for inclusion in Meta-analysis 1** \| **Result available for inclusion in Meta-analysis 2** \| **Result available for inclusion in Meta-analysis 3** \| **Result available for inclusion in Meta-analysis 4** \| **Result available for inclusion in Meta-analysis 5** \| \| --- \| --- \| --- \| --- \| --- \| --- \| --- \| --- \| \| Fangfang Li 2023 \| PMID: 37598237 \| 64 \| ✓ \| ~ \| ✓ \| ~ \| ~ \| \| Grønhøj 2018 \| PMID: 29678523 \| 30 \| ✓ \| ~ \| ✓ \| ~ \| ✓ \| \| Lynggaard 2022 \| PMID: 35486613 \| 30 \| ~ \| ✓ \| ~ \| ✓ \| ~ \| \| Jakobsen 2024 \| PMID: 38441659 \| 120 \| ✓ \| ~ \| ✓ \| ~ \| ✓ \| \| Carlander 2024 \| PMID: 39751638 \| 120 \| ~ \| ✓ \| ~ \| ✓ \| ~ \| |

| **Step 3. Consider the potential for missing studies across the systematic review** |
| --- |
| **Answer the following questions to determine whether circumstances indicate potential for some eligible studies not being identified because of the P value, magnitude or direction of the results generated. Answer these questions once, in relation to the systematic review as a whole.**   \| **Question** \| **Response options** \| \| --- \| --- \| \| **3.1. Were prospectively registered studies or studies identified for a prospective meta-analysis the only type of study eligible for inclusion in the review?** \| Y \| \| **3.2. If N to 3.1: Would you expect every eligible study to be identifiable regardless of its results?** \| NA \| \| **3.3. If Y/PY to 3.2: Were you likely to have found all eligible studies regardless of their results?** \| PY \|   Y: ‘Yes’; PY: ‘Probably yes’; PN: ‘Probably no’; N: ‘No’; NA: ‘Not applicable’.  **Check the box below if the response to 3.1 was ‘No’ and the response to 3.2 or 3.3 was ‘No / Probably no’**  Circumstances indicate potential for some eligible studies not being identified because of the P value, magnitude or direction of the results generated  **Provide any relevant information to support responses**   \|  \| \| --- \| |

**Step 4. Assess risk of bias due to missing evidence in a meta-analysis (complete for each meta-analysis)**

Responses underlined in green are potential markers for low risk of bias, and responses in red are potential markers for a risk of bias.

| **Details of the meta-analysis being assessed for risk of bias** | | |
| --- | --- | --- |
| **Specify the meta-analysis** | 1 | |
| **Specify the meta-analysis result (e.g. summary effect estimate and 95% CI)** | The MD was 0.06 ml/min (95%CI: -0.05 to 0.17), favoring MSC treatment. The 95% prediction interval ranged from -0.15 to 0.26. | |
| **Specify the number of included studies and participants** | 3 studies and 214 participants included | |
| **Risk of bias assessment** | | |
| **Signalling questions** | **Comments** | **Response options** |
| ***The following questions relate to the within-study assessment of non-reporting bias (‘known unknowns’)*** | | |
| **4.1. Of the studies identified, was there any for which no result was available for inclusion in the meta-analysis, likely because of the P value, magnitude or direction of the result generated (refer to Step 2)?** |  | N |
| **4.2. If Y to 4.1: Is it likely that there would be a notable change to the summary effect estimate if the omitted results had been included?** |  | NA |
| **4.3. Of the studies identified, was there any for which it was unclear whether an eligible result was generated (refer to Step 2)?** |  | N |
| **4.4. If Y to 4.3: Is it likely that there would be a notable change to the summary effect estimate if the potentially omitted results had been included?** |  | NA |
| ***The following questions relate to the across-study assessment of non-reporting bias (‘unknown unknowns’)*** | | |
| **4.5 Do circumstances (identified in Step 3) indicate potential for some eligible studies not being identified because of the P value, magnitude or direction of the results generated?** |  | N |
| **4.6. If Y to 4.5: Is it likely that studies not identified had results that were eligible for inclusion in the meta-analysis?** |  | NA |
| **4.7. If Y to 4.1, 4.3 or 4.5: Does the pattern of observed study results suggest that the meta-analysis is likely to be missing results that were systematically different (in terms of P value, magnitude or direction) from those observed?** |  | NA |
| **4.8. If Y/PY/NI to 4.2, 4.4, 4.6 or 4.7: Did sensitivity analyses suggest that the summary effect estimate was biased due to missing results?** |  | NA |
| **Risk of bias judgement** |  | Low |
| Optional: What is the predicted direction of bias for this meta-analysis? | If any risk of bias is present, it is estimated to favour the experimental treatment. | Favours experimental |

Y: ‘Yes’; PY: ‘Probably yes’; PN: ‘Probably no’; N: ‘No’; NI: ‘No information’; NA: ‘Not applicable’.

| **Details of the meta-analysis being assessed for risk of bias** | | |
| --- | --- | --- |
| **Specify the meta-analysis** | 2 | |
| **Specify the meta-analysis result (e.g. summary effect estimate and 95% CI)** | The MD was 0.01 ml/min (95%CI: -0.02 to 0.05) | |
| **Specify the number of included studies and participants** | 2 studies and 150 participants included | |
| **Risk of bias assessment** | | |
| **Signalling questions** | **Comments** | **Response options** |
| ***The following questions relate to the within-study assessment of non-reporting bias (‘known unknowns’)*** | | |
| **4.1. Of the studies identified, was there any for which no result was available for inclusion in the meta-analysis, likely because of the P value, magnitude or direction of the result generated (refer to Step 2)?** |  | N |
| **4.2. If Y to 4.1: Is it likely that there would be a notable change to the summary effect estimate if the omitted results had been included?** |  | NA |
| **4.3. Of the studies identified, was there any for which it was unclear whether an eligible result was generated (refer to Step 2)?** |  | N |
| **4.4. If Y to 4.3: Is it likely that there would be a notable change to the summary effect estimate if the potentially omitted results had been included?** |  | NA |
| ***The following questions relate to the across-study assessment of non-reporting bias (‘unknown unknowns’)*** | | |
| **4.5 Do circumstances (identified in Step 3) indicate potential for some eligible studies not being identified because of the P value, magnitude or direction of the results generated?** |  | N |
| **4.6. If Y to 4.5: Is it likely that studies not identified had results that were eligible for inclusion in the meta-analysis?** |  | NA |
| **4.7. If Y to 4.1, 4.3 or 4.5: Does the pattern of observed study results suggest that the meta-analysis is likely to be missing results that were systematically different (in terms of P value, magnitude or direction) from those observed?** |  | NA |
| **4.8. If Y/PY/NI to 4.2, 4.4, 4.6 or 4.7: Did sensitivity analyses suggest that the summary effect estimate was biased due to missing results?** |  | NA |
| **Risk of bias judgement** |  | Low |
| Optional: What is the predicted direction of bias for this meta-analysis? | If any risk of bias is present, it is estimated to favour the experimental treatment. | Favours experimental |

| **Details of the meta-analysis being assessed for risk of bias** | | |
| --- | --- | --- |
| **Specify the meta-analysis** | 3 | |
| **Specify the meta-analysis result (e.g. summary effect estimate and 95% CI)** | The MD was 0.05 ml/min (95%CI -0.32 to 0.42) | |
| **Specify the number of included studies and participants** | 3 studies and 214 participants included | |
| **Risk of bias assessment** | | |
| **Signalling questions** | **Comments** | **Response options** |
| ***The following questions relate to the within-study assessment of non-reporting bias (‘known unknowns’)*** | | |
| **4.1. Of the studies identified, was there any for which no result was available for inclusion in the meta-analysis, likely because of the P value, magnitude or direction of the result generated (refer to Step 2)?** |  | N |
| **4.2. If Y to 4.1: Is it likely that there would be a notable change to the summary effect estimate if the omitted results had been included?** |  | NA |
| **4.3. Of the studies identified, was there any for which it was unclear whether an eligible result was generated (refer to Step 2)?** |  | N |
| **4.4. If Y to 4.3: Is it likely that there would be a notable change to the summary effect estimate if the potentially omitted results had been included?** |  | NA |
| ***The following questions relate to the across-study assessment of non-reporting bias (‘unknown unknowns’)*** | | |
| **4.5 Do circumstances (identified in Step 3) indicate potential for some eligible studies not being identified because of the P value, magnitude or direction of the results generated?** |  | N |
| **4.6. If Y to 4.5: Is it likely that studies not identified had results that were eligible for inclusion in the meta-analysis?** |  | NA |
| **4.7. If Y to 4.1, 4.3 or 4.5: Does the pattern of observed study results suggest that the meta-analysis is likely to be missing results that were systematically different (in terms of P value, magnitude or direction) from those observed?** |  | NA |
| **4.8. If Y/PY/NI to 4.2, 4.4, 4.6 or 4.7: Did sensitivity analyses suggest that the summary effect estimate was biased due to missing results?** |  | NA |
| **Risk of bias judgement** |  | Low |
| Optional: What is the predicted direction of bias for this meta-analysis? | If any risk of bias is present, it is estimated to favour the experimental treatment. | Favours experimental |

| **Details of the meta-analysis being assessed for risk of bias** | | |
| --- | --- | --- |
| **Specify the meta-analysis** | 4 | |
| **Specify the meta-analysis result (e.g. summary effect estimate and 95% CI)** | The MD was -0.04 mL/min (95% CI: -0.17 to 0.10) | |
| **Specify the number of included studies and participants** | 2 studies and 150 participants included | |
| **Risk of bias assessment** | | |
| **Signalling questions** | **Comments** | **Response options** |
| ***The following questions relate to the within-study assessment of non-reporting bias (‘known unknowns’)*** | | |
| **4.1. Of the studies identified, was there any for which no result was available for inclusion in the meta-analysis, likely because of the P value, magnitude or direction of the result generated (refer to Step 2)?** |  | N |
| **4.2. If Y to 4.1: Is it likely that there would be a notable change to the summary effect estimate if the omitted results had been included?** |  | NA |
| **4.3. Of the studies identified, was there any for which it was unclear whether an eligible result was generated (refer to Step 2)?** |  | N |
| **4.4. If Y to 4.3: Is it likely that there would be a notable change to the summary effect estimate if the potentially omitted results had been included?** |  | NA |
| ***The following questions relate to the across-study assessment of non-reporting bias (‘unknown unknowns’)*** | | |
| **4.5 Do circumstances (identified in Step 3) indicate potential for some eligible studies not being identified because of the P value, magnitude or direction of the results generated?** |  | N |
| **4.6. If Y to 4.5: Is it likely that studies not identified had results that were eligible for inclusion in the meta-analysis?** |  | NA |
| **4.7. If Y to 4.1, 4.3 or 4.5: Does the pattern of observed study results suggest that the meta-analysis is likely to be missing results that were systematically different (in terms of P value, magnitude or direction) from those observed?** |  | NA |
| **4.8. If Y/PY/NI to 4.2, 4.4, 4.6 or 4.7: Did sensitivity analyses suggest that the summary effect estimate was biased due to missing results?** |  | NA |
| **Risk of bias judgement** |  | Low |
| Optional: What is the predicted direction of bias for this meta-analysis? | If any risk of bias is present, it is estimated to favour the experimental treatment. | Favours experimental |

| **Details of the meta-analysis being assessed for risk of bias** | | |
| --- | --- | --- |
| **Specify the meta-analysis** | 5 | |
| **Specify the meta-analysis result (e.g. summary effect estimate and 95% CI)** | The MD was 0.03 (95% CI: 0.01 to 0.05) | |
| **Specify the number of included studies and participants** | 2 studies and 150 participants included | |
| **Risk of bias assessment** | | |
| **Signalling questions** | **Comments** | **Response options** |
| ***The following questions relate to the within-study assessment of non-reporting bias (‘known unknowns’)*** | | |
| **4.1. Of the studies identified, was there any for which no result was available for inclusion in the meta-analysis, likely because of the P value, magnitude or direction of the result generated (refer to Step 2)?** |  | N |
| **4.2. If Y to 4.1: Is it likely that there would be a notable change to the summary effect estimate if the omitted results had been included?** |  | NA |
| **4.3. Of the studies identified, was there any for which it was unclear whether an eligible result was generated (refer to Step 2)?** |  | N |
| **4.4. If Y to 4.3: Is it likely that there would be a notable change to the summary effect estimate if the potentially omitted results had been included?** |  | NA |
| ***The following questions relate to the across-study assessment of non-reporting bias (‘unknown unknowns’)*** | | |
| **4.5 Do circumstances (identified in Step 3) indicate potential for some eligible studies not being identified because of the P value, magnitude or direction of the results generated?** |  | N |
| **4.6. If Y to 4.5: Is it likely that studies not identified had results that were eligible for inclusion in the meta-analysis?** |  | NA |
| **4.7. If Y to 4.1, 4.3 or 4.5: Does the pattern of observed study results suggest that the meta-analysis is likely to be missing results that were systematically different (in terms of P value, magnitude or direction) from those observed?** |  | NA |
| **4.8. If Y/PY/NI to 4.2, 4.4, 4.6 or 4.7: Did sensitivity analyses suggest that the summary effect estimate was biased due to missing results?** |  | NA |
| **Risk of bias judgement** |  | Low |
| Optional: What is the predicted direction of bias for this meta-analysis? | If any risk of bias is present, it is estimated to favour the experimental treatment. | Favours experimental |
